# The “sacral parasympathetic”: ontogeny and anatomy of a myth

**DOI:** 10.1007/s10286-017-0478-7

**Published:** 2017-11-04

**Authors:** Isabel Espinosa-Medina, Orthis Saha, Franck Boismoreau, Jean-François Brunet

**Affiliations:** Institut de Biologie de l’ENS (IBENS), INSERM, CNRS, École Normale Supérieure, PSL Research University, 75005 Paris, France

**Keywords:** Anatomy, Parasympathetic neurons, Pelvic ganglion, Sacral parasympathetic nucleus, Sympathetic neurons

## Abstract

We recently defined genetic traits that distinguish sympathetic from parasympathetic neurons, both preganglionic and ganglionic (Espinosa-Medina et al., Science 354:893–897, 2016). By this set of criteria, we found that the sacral autonomic outflow is sympathetic, not parasympathetic as has been thought for more than a century. Proposing such a belated shift in perspective begs the question why the new criterion (cell types defined by their genetic make-up and dependencies) should be favored over the anatomical, physiological and pharmacological considerations of long ago that inspired the “parasympathetic” classification. After a brief reminder of the former, we expound the weaknesses of the latter and argue that the novel genetic definition helps integrating neglected anatomical and physiological observations and clearing the path for future research.

## Introduction

**Fig. 1:**
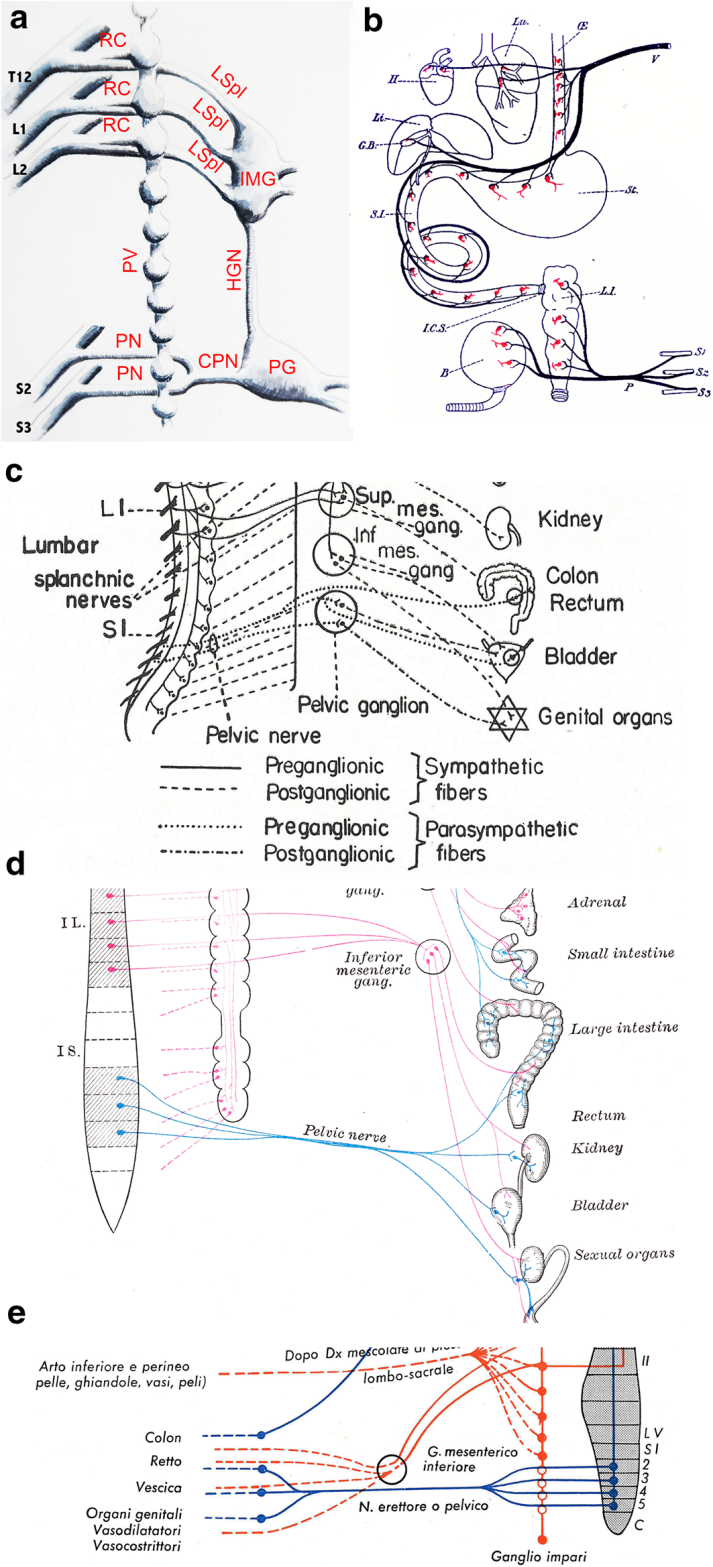
Schematics of the autonomic innervation of the pelvis. **a** Schematic of the projection of the spinal cord (spinal nerves T12, L1, L2, S2 and S3) to the lumbar and sacral autonomic ganglia. The pelvic ganglion receives a dual input, thoraco-lumbar through the hypogastric nerve, and sacral from the pelvic nerves. The sacral paravertebral chain does not receive projections (*rami communicantes*) from the sacral cord. Abbreviations: HGN hypogastric nerve, IMG inferior mesenteric ganglion, PG pelvic ganglion, PN pelvic nerve, CPN common pelvic nerve, LSpl lumbar splanchnic nerves, RC ramus communicans, SC spinal cord, PV paravertebral chain. **b** Schematic of the cranio-sacral division by Gaskell [11]: no pelvic ganglion is shown, and the extra-mural neurons postganglionic to the pelvic nerves are represented exactly like the intramural ones postganglionic to the vagus nerve. **c** Kuntz [31] represents the pelvic ganglion as a purely parasympathetic relay, devoid of lumbar connection, 10 years after his own description of a dual input [5]. **d** The 24th edition of* Gray’s anatomy* (1948) omits the pelvic ganglion on the path of the pelvic nerve. This is unchanged in recent editions [9]. **e** Testut and Latarjet [8] [Italian translation (1971) of the 9th French edition (1949)] omit the pelvic ganglion and represent (in blue) scattered distal relays of the sacral preganglionics. Contemporary printed or online schematics display variations on these features (e.g. [7])

Neurons in the sympathetic and parasympathetic ganglia receive input from preganglionic neurons located in the central nervous system (CNS). Sympathetic preganglionics are in the intermediate zone of the thoracolumbar spinal cord. Parasympathetic preganglionics are in nuclei or loose neuronal aggregates of the brainstem and, as claimed for more than a century, in the intermediolateral column of the sacral spinal cord, where they form the “sacral parasympathetic nucleus.” The assignation of the sacral outflow to the parasympathetic division of the autonomic nervous system was introduced at the turn of the twentieth century by Langley, in a remarkably cursory fashion, with a brief justification in 1899 [2], followed by reaffirmation in footnotes to two articles published in 1905 [3] (p. 403) (“I use the word para-sympathetic for the cranial and sacral autonomic systems”) and 1911 [4] (p. 173) (a schematic, introduced by “I divide the nervous system as follows:”). It was never challenged since. Later, when the pelvic ganglion (improperly called “plexus”—which better describes a mere rearrangement of fibers—in species where it is more diffuse) was recognized as receiving both thoracolumbar and sacral inputs (through, respectively, the hypogastric and pelvic nerves) [5, 6] (Fig. 1a), it was deemed to be of a mixed sympathetic/parasympathetic nature, a unique case among autonomic ganglia. Possibly because of this complication, standard schematics of the autonomic nervous system omit either the pelvic ganglion altogether or its sympathetic input [7–9] (Fig. 1b–e). Nevertheless, the anatomical convergence in the pelvic ganglion—and projection from there to the viscera—of a sympathetic outflow from the lumbar level and of a supposedly parasympathetic one from the sacral level has represented the framework for the study of pelvic physiology at least since the 1930s.

## A genetic viewpoint

We recently defined developmental genetic signatures that distinguish sympathetic from parasympathetic neurons and assessed their status in the sacral outflow of the mouse [1]. We found that sacral preganglionic neurons, like thoracolumbar ones and unlike cranial ones, depend on the basic loop–helix–loop transcription factor *Olig2* but not on the homeodomain transcription factor *Phox2b*, and that they express *Foxp1* but not *Phox2a*, *Tbx2*, *Tbx3* or *Tbx20*. Sacral preganglionic neurons thus have the hallmarks of genuine sympathetic neurons. Likewise, all neurons in the pelvic ganglion, as well as intramural ganglia of the bladder, like sympathetic ones and unlike parasympathetic ones, express the transcription factors *Islet1*, *Gata3* and *Hand1* but neither *Hmx2* nor *Hmx3*, and they develop independently of their afferent nerves. Thus, by the criterion of cell type, the neurons of the autonomic nervous system in the sacral spinal cord and in the pelvic ganglion are all sympathetic. This conclusion clashes with the traditional view, which is based on a variety of arguments that we critically review below.

## The anatomical arguments

### The lumbar gap, the white *ramus communicantes* and the *nervi erigentes*

At a time when the autonomic nervous system was still considered to be entirely “sympathetic”, foreshadows of the “sacral parasympathetic” outflow can be spotted in the parallel drawn by Walter Gaskell between the cranial and sacral visceral nerves. He first described [10] that the nerves from the CNS to autonomic ganglia form a continuous series at thoracic and upper lumbar levels, framed by two gaps at brachial and lower lumbar levels and then resuming below at sacral levels as projections to the pelvic ganglia and above at cranial levels as projections of the vagus nerve to “distal” ganglia (that Gaskell changed his mind about: “ganglia of the trunci vagi and ganglion petrosum” in 1885 [10] (p. 11) give way to “excitor neurons of the main viscera” in 1920 [11]). Thus, the cranial and sacral outflows symmetrically bracket, at a distance, the thoracolumbar one. Gaskell acknowledges that the parallel between the brachial and lumbar gaps is “rough” [11] (p 27). This parallel is actually  false: the caudal gap coincides with the lumbar plexus (and might be explained by an ontogenetic depletion of preganglionic neurons in favor of somatic motor neurons for the hindlimb, both born from the same pool of progenitors [12]). In contrast, the rostral gap extends beyond the brachial plexus to include the entire cervical spinal cord.

**Fig. 2:**
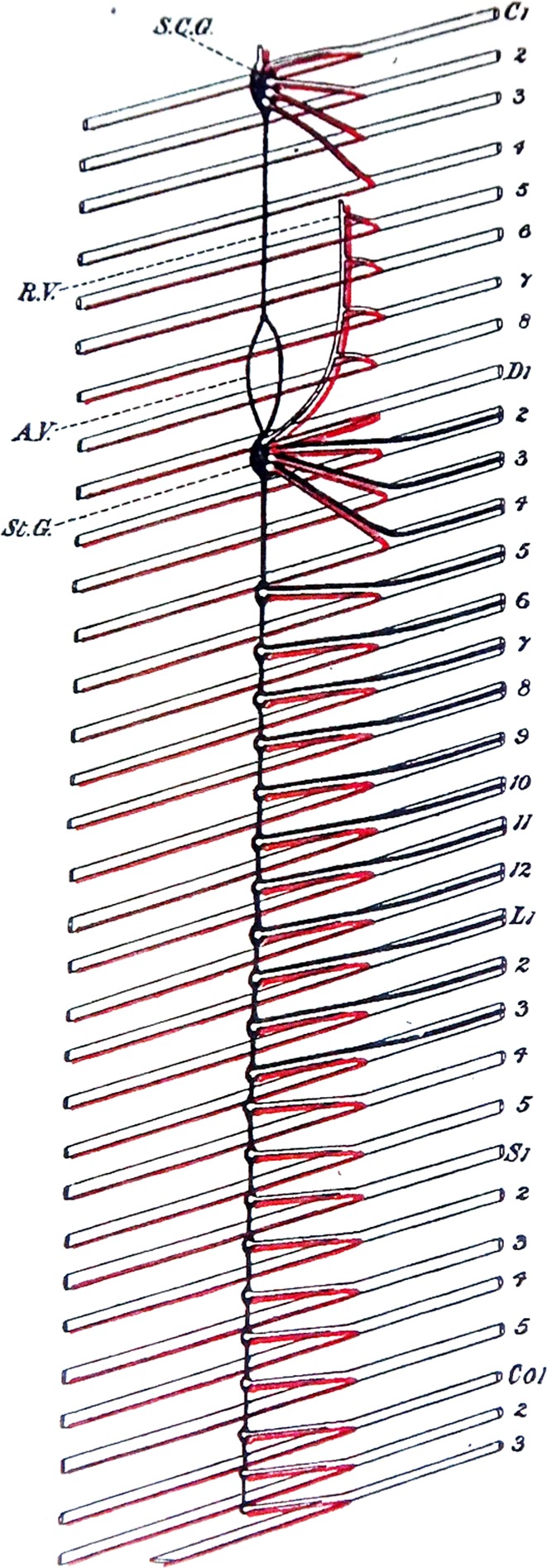
Connections between the spinal cord and the sympathetic chain, as originally described by Gaskell: Fig. 4 from [11] showing (in black) the white *ramus comunicans* from the spinal cord (which would be on the right of the figure, not shown) through the ventral roots of the second thoracic (D2) to third lumbar (L3) nerves, but neither rostrally nor caudally from there. Neither the superior cervical ganglion (S.C.G.) nor the sacral ones (S1 and below) receive input from their rostro-caudal level, but rather from the thoracolumbar cord below or above, respectively, via longitudinal projection through the paravertebral chain. The projections from sympathetic ganglia to the spinal nerves (grey *rami communicans*) are shown in red. A.V. annulus of Vieussens (or subclavian loop), R.V. ramus vertebralis, St.G. stellate ganglion

**Fig. 3:**
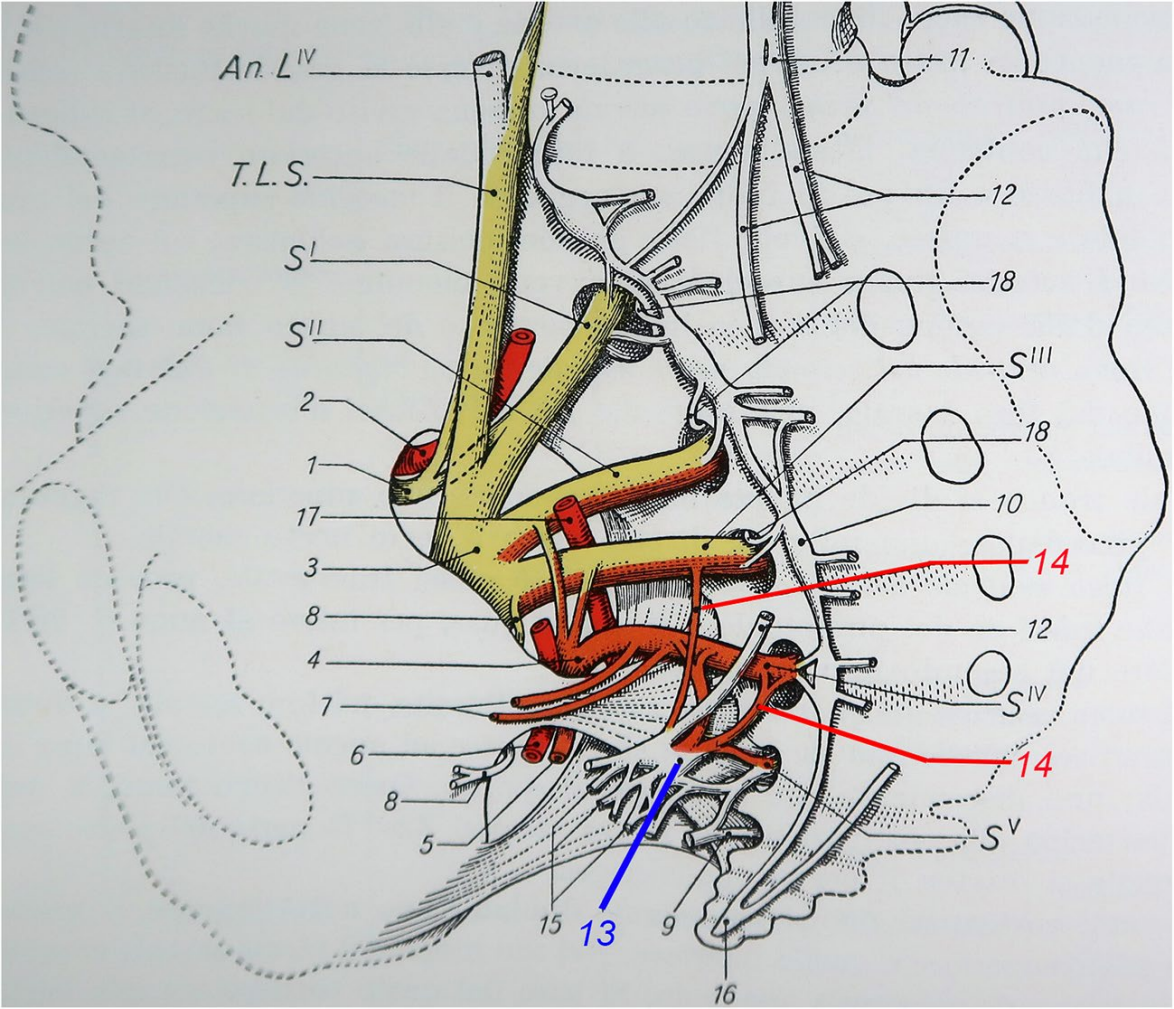
Semi-schematic representation of the pelvic nerves from Testut and Latarjet, 9th edition [8]: the pelvic nerves (labeled 14, in red] emanating from the third and fourth sacral nerves (S^III^ and S^IV^) directly project to the pelvic ganglion (labeled 13, in blue) while completely bypassing the sacral paravertebral chain (labeled 10 and connected to the spinal nerves by one or two grey *rami communicantes*, unlabeled on the figure). Other numbers refer to the coccygeal plexus (9), presacral nerve (anterior hypogastric plexus) (11), hypogastric nerves (12), efferent branches of the pelvic ganglion (15), ganglion of Walther (ganglion impar) (16), branch of sacral sympathetic contributing to the meningeal nerve (18)

A second “cranio-sacral” parallel suggested by Gaskell was that, above the brachial and below the lumbar gaps, projections resume only to distal or “collateral” ganglia, not to the paravertebral sympathetic chain; in other words, there are no white *rami communicantes* [10] (Figs. 1a, 2). Again, the parallel is superficial: there is no paravertebral chain at the cranial level, so that the absence of a white *ramus communicans* there is trivial; in contrast, there are sacral paravertebral ganglia, however diminutive, so that the absence of this connection at sacral levels is a genuine oddity. The pelvic ganglion is connected to branches of the spinal nerves that bypass the paravertebral chain, the *nervi erigentes* of Eckhard [13], which can appear to the anatomist as unrelated to anything at thoracolumbar levels (Fig. 3). (According to Langley [14] (p. 234), these nerves had already been divorced from the “great sympathetic” by J.-B. Winslow in the 1730s). However, Gaskell was keen, at first, to emphasize the kinship of the *nervi erigentes* to the “abdominal splanchnics” (that connect the thoracolumbar preganglionics with the prevertebral—coeliac and mesenteric—ganglia “without having anything to do with the [paravertebral] chain”) [11] (p. 24)—even though they join it at gross anatomical level (Fig. 1a); to mark this similarity he originally named the *nervi erigentes* “pelvic splanchnic” [10], but he eventually backtracked and validated Langley’s “pelvic nerves” [11] (p. 25). The fact that sacral paravertebral ganglia receive their spinal input not from the nearby sacral cord but from the lumbar cord via the sympathetic chain needs an explanation, which might reside in a temporal or spatial idiosyncrasy in the development of this caudal region.

### Proximity to target organs

An often-cited differential feature of sympathetic and parasympathetic ganglia is their distance to their targets, long and short, respectively. This rule, however, is soft and implicitly excludes prevertebral ganglia (sympathetic yet close to the gut, kidneys, etc.). The pelvic ganglia, which are supposedly mixed, inevitably conflict with this criterion: they are so close to the bladder and internal genital organs as to have spawned the category of “short noradrenergic [i.e. sympathetic] neurons” [15] and so distant from external genitals as to create the unusual danger of accidental or surgical disruption of nerves between a supposedly parasympathetic ganglion and its target [16].

### Anatomical counterarguments

Conversely, two anatomical features could have inspired long ago a grouping, as we propose, of all spinal preganglionic neurons and their distinction from cranial ones:

1. All spinal preganglionics—thoracolumbar and sacral—project out of the CNS through the ventral spinal roots, together with somatic motor neurons, whereas cranial (bulbar) ones have a dedicated dorsolateral exit point. The reasons why Gaskell, in his fastidious anatomical enquiries, did not ponder this blatant feature may be that: (1) he originally thought that projections to the sympathetic chains traveled through both the ventral and dorsal roots [10] (p. 4); (2) he and others saw cranial preganglionics neurons as equivalent, or serial homologs of the spinal ones—both were subsumed under the term “general visceral efferent,” a parallel still rampant in contemporary treatments of the subject (e.g. [17])—which they are not. A falsely unified vision of the cranial and spinal visceral outflows seems to have paved the way for their later false partition into thoracolumbar and cranio-sacral.
2. On the sacral autonomic path to the gut, the presynaptic partners of enteric neurons are in the peripheral nervous system (in pelvic ganglia), as they are on the thoracolumbar path (in mesenteric ganglia) [18, 19], whereas on the cranial path they are in the CNS (in the dorsal motor nucleus of the vagus nerve). Gaskell invented the prototype [11] (Fig. 1b) of a long series of ambiguous or outright erroneous drawings (Fig. 1d, e) that, to this day, blur the difference. Probably in the hope of reducing this discrepancy between sacral and cranial “parasympathetics”, direct projections from the sacral cord to enteric ganglia have been sought: they were found to be absent [18] or rare [19] (and in the latter case, in equal numbers in the sacral and thoracolumbar cords).

A more recent anatomical counterargument to the sacral “parasympathetic” label is that a number of individual pelvic ganglionic neurons receive dual lumbosacral innervation (which undermines the hodological definition of sympathetic versus parasympathetic ganglionic neurons), as inferior mesenteric ganglionic neurons do (which undermines the singularity of the pelvic ganglion) [6].

In sum, there are more anatomical arguments against than in favor of a parasympathetic identity of the sacral autonomic outflow.

## The pharmacological argument

The sensitivity of “receptive substances” to agonists and antagonists played a major role in consolidating Langley’s proposal of the sacral and cranial “as one system” (thus “parasympathetic”): several physiological responses to the stimulation of pelvic nerves are mimicked by the muscarinic receptor agonist pilocarpine (but not by adrenaline), just like salivation or myosis—genuinely parasympathetic effects—reflecting the fact that pelvic ganglia contain many cholinergic neurons [16]. However, this pharmacological or neurotransmitter criterion logically should have been dropped as early as 1934 when Dale and Feldberg found the sympathetic innervation to sweat glands to be cholinergic [20] (and pilocarpine causes sweating). By then though, 10 years after Langley’s crowning opus [21]—and despite many cautionary notes about gaps in the “theories on the relation of drugs to nerve systems” (p. 39)—the dogma had solidified.

Cholinergic neurons in both para- and prevertebral sympathetic chains are well described (reviewed in [22]): they innervate sweat glands, the periosteum [23] and muscle arteries in non-primates (reviewed in [24]). Hodologically defined pelvic “parasympathetic” neurons and cholinergic ones only partially overlap [16], and some pelvic “parasympathetic” neurons express tyrosine hydroxylase and thus synthesize noradrenaline [25]. Yet, to this day, the old “cholinergic” argument occasionally resurfaces [26] in a feeble echo of a long gone past.

## The physiological arguments

Another major support for a “parasympathetic sacral” outflow stems from a generalization of Gaskell’s distinction between “anabolic” and “catabolic” fibers to the heart, and the ensuing notion of a lumbosacral antagonism on pelvic function. We discuss below the prominent cases of micturition and erection.

### Micturition

**Fig. 4:**
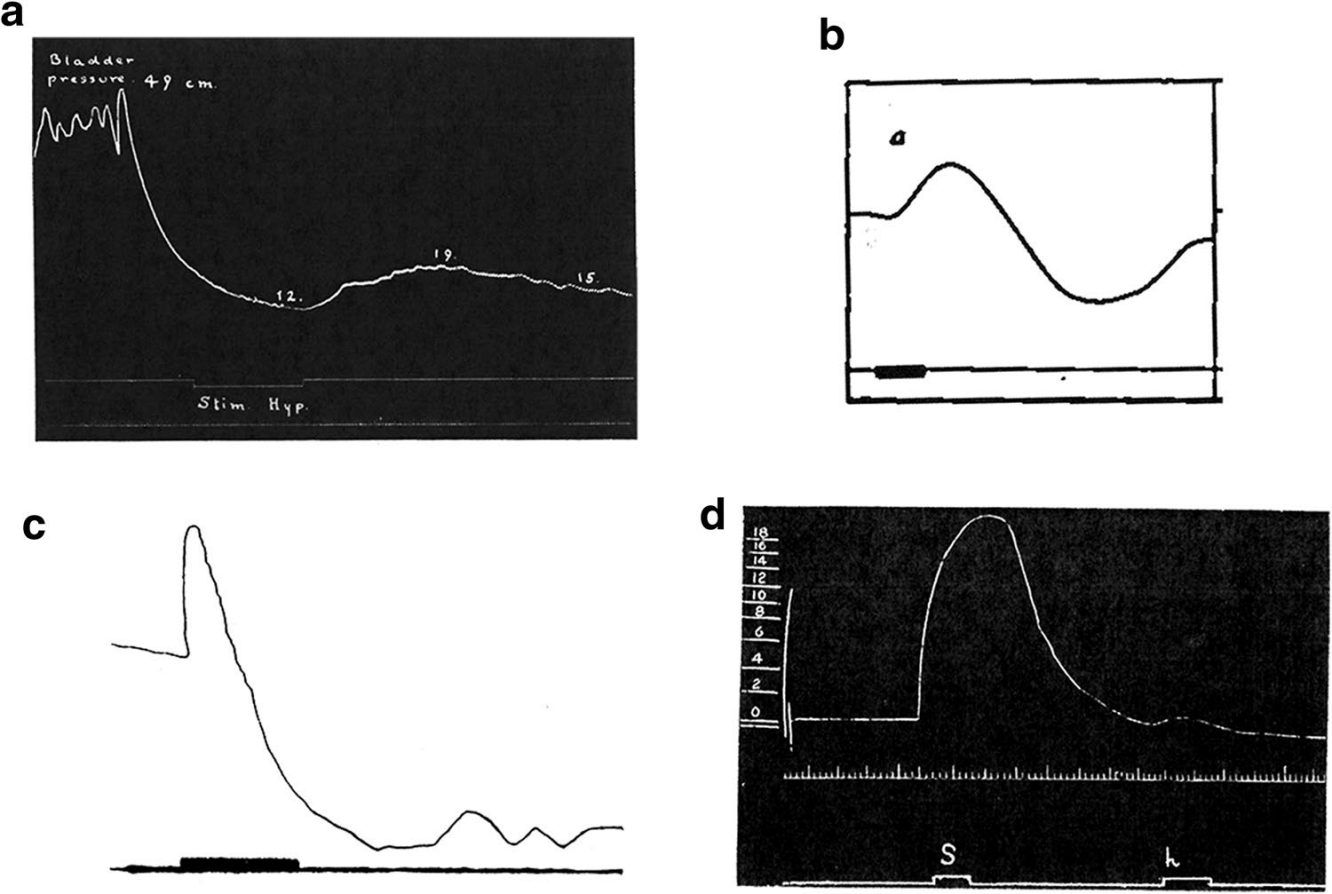
Response of the bladder to stimulation of the hypogastric nerve according to **a** Elliott [29] in cats (but not most other species), isometric pressure record. Note that “as the lever writes on an arc of great curvature, at the top of the tracing it is much to the left of the vertical line through the signal”; **b** Stewart [30], in cat, kymographic record, bar: 10 s; **c** Kuntz, kymographic record (reproduced in [31], bar: 40 s;** d** Fagge [32], in dog, isometric pressure curve, stimulation of the pelvic nerve (s) and hypogastric (h), the latter eliciting only a slight contraction

In the field of pelvic physiology, few topics are more confusing than the lumbar inhibitory pathway to the detrusor muscle of the bladder. Langley himself never used the bladder to make his case for the sympatho-parasympathetic duality of the lumbo-sacral outflow. And for good reason: he had explicitly excluded any inhibition of the bladder in his 1895 extensive monography on the subject [27]: “We give some additional—and we think conclusive—evidence that both the lumbar and the sacral nerves cause contraction of all the muscle fibers of the bladder […]. Inhibitory fibers of the bladder are few, if indeed they exist.”. Three years earlier, Sherrington had published data on monkey and cat [28] that “confirmed […] the existence of a motor outflow [to the bladder] in the upper lumbar anterior roots”, and, without mentioning any inhibition, plainly formulated the continuity of action between the lumbar and sacral levels: “we may suppose that a long nucleus for the bladder exists in the lumbosacral region, which has however a gap in its continuity […]. The outflow from the anterior roots above the gap is into the sympathetic system, from the anterior roots below the gap is direct by the sacral nerves. To a suggestion as to the wherefore of this gap I hope to return later” (which he apparently forgot to do). Elliott [29], following Stewart [30], contradicted Langley on the subject concerning the cat and monkey, yet failed to detect lumbar inhibition in all other species he tested: dog, rabbit, ferret, Indian mongoose and Indian civet—except, very tentatively, in the goat. Moreover, in his opinion, the human bladder failed to respond to being “painted” with noradrenaline. A feature of the bladder response to stimulation of the hypogastric nerves is that whenever a relaxation occurs, it is only apparent, without exception, tens of seconds after an initial contraction, comparable in timing to the contraction evoked by pelvic nerves (and possibly related to the contraction of the base and trigone of the bladder (discussed in [31], p. 295). Langley himself noted [27] that “a slight flaccidity follows the contraction brought about by the hypogastrics, but we never observed the flaccidity without the contraction, nor any considerable degree of flaccidity.” The disagreement seems to bear on the interpretation of this sluggish and inconstant secondary response (the main published traces we are aware of are reproduced in Fig. 4). Langworthy is possibly the second major provider of evidence for an inhibitory action of the lumbar pathway on the bladder, by demonstrating, in cats, a diminished vesical capacity after hypogastric nerve transection [33]. Yet, he later failed to find any sympathetic fibers in the detrusor muscle [34] (followed in this by many others [35–38]) and eventually turned into the bluntest critic of the lumbosacral antagonism on the bladder [39]: “Section of the sympathetic fibers has no significant effect on the urinary bladder, which acts as well as ever. Section of the parasympathetic fibers paralyzes the bladder completely.” De Groat et al. [40] (also on cat) is a later notable contribution to the “lumbar inhibition” case. The paper, however, is introduced by a warning that “a considerable body of earlier data [indicates] that the sympathetic pathways [are] relatively unimportant in the regulation of bladder function” and closes on the concession that “we have been unable to demonstrate that […] depression of the detrusor […] is activated by naturally occurring sympathetic firing”. Intense firing of the hypogastric nerve occurs during micturition [41], an observation rarely if ever cited, possibly because it is more suggestive of synergy rather than antagonism of the lumbar and sacral pathways. Rare patients with a genetic deficiency for dopamine-β-hydroxylase, and thus unable to synthesize noradrenaline, have normal urinary function [42] and studies in humans with lesions in the spinal cord [43] make it “unlikely that spinal reflexes governing lower urinary tract function in man include the sympathetic [i.e. lumbar] nervous system”—i.e. either the proposed relaxation of the detrusor or the proposed contraction of the bladder outlet by the thoracolumbar outflow [44]. Yet all contemporary reviews and textbooks on the subject, particularly in their take-home schematics, portray a well-balanced antagonism between the lumbar pathway and the sacral one during bladder filling and micturition (e.g. [44, 45]).

In this monotonously discordant literature that covers more than a century, there is no sign of a final convergence of the reviews, textbooks and primary literature. We propose that this stalemate is caused by the double bind of the “sacral parasympathetic” dogma, with its implication of a lumbosacral antagonism, and the scarcity of evidence for the latter. The shift towards purely pharmacological experiments in the 1970s, highlighting alpha- and beta-adrenergic receptors in different parts of the bladder and under different physiopathological conditions, is built on the notion of a sympathetic lumbar inhibitory pathway without providing further evidence for its reality. Local non-neuronal sources of adrenalin have been recently proposed [38].

### Erection

If bladder physiology did little, originally, to help theorizing the sacral parasympathetic outflow, blood vessels were the crux of the matter. In his 1899 Presidential Address to the Physiological Society [2], credited by Langley himself [21] as his first published statement on “the sacral and cranial pathways as one system”, he puts forward “one reason” to support this view: “if regions above and below [the middle portion of the spinal cord] were mere separated parts of the sympathetic region we should expect that, when some of these regions and the sympathetic region send nerves to the same spots, the effects produced by both sets of nerves would be the same in kind though it might differ in extent. But it is often not the case. Thus, certain blood vessels may receive nerve fibers from four spinal nerves in the sympathetic region and three spinal nerves in the sacral region, all the former cause contraction of the blood vessels, all the latter cause dilatation”. Prominent among the “certain blood vessels” are those to the external genitals, analyzed by Langley and Anderson [27], that cause erection by vasodilation and detumescence by vasoconstriction. On this issue, however, Langley was and remained somewhat of an outsider. Twenty years earlier, Eckhard had found [13] that “in the rabbit, the mechanism of erection is provided by nerves via a second pathway, that is by the nerve trunk that anatomically corresponds to the superior hypogastric plexus in man [i.e. the hypogastric nerve]”.^1^ In 1895, the same year as Langley and Anderson’s paper, François-Franck announced that he “obtained a penile vasodilation from the descending branches of the inferior mesenteric ganglion with the same clarity as with the common sacral erector nerve”^2^ [46]. He also found an occasional vasoconstrictor effect of the anterior pelvic nerve and concluded that “with the exception of the posterior erector nerve of Eckhard one finds associated in all nerves vasoconstrictor and vasodilator fibers”, “whose combined effect depended on the “nature form, intensity and rhythm of the excitations”. Half a century later, a similar explanation (“a masking effect produced by concurrent stimulation of vasoconstrictor fibers”) was offered by Root and Bard [47] for the failure of Langley to see “the suprasacral vasodilator pathway to the penis [that] runs through sympathetic channels”, that they deduced from a series of 154 tests on 21 cats with precise spinal lesions. Bessou and Laporte [48] concluded their own study on cat by a similar observation: “The hypogastric nerve, belonging to the orthosympathetic system, contain, in the cat, cholinergic vasodilatating fibers whose prolonged and repetitive stimulation can provoke an erection, despite the presence of fibers with an opposing action”.^3^ Human patients with complete destruction of the lower lumbar or sacral cord, but not the upper lumbar or thoracic one, experience psychogenic erections mediated by the lumbar pathway [49, 50]. In 1979, Sjöstrand and Klinge found that the pelvic and hypogastric outflows are synergistically vasodilatory in rabbit [51] and commented, with a hint of annoyance: “We feel that now, more than a hundred years after their original description (referring to [13]), it is time to generally accept the existence of the sympathetic hypogastric erectile fibers.” However, 40 years later, the time for “general acceptance” has yet to come and, here again, we incriminate the conceptual sway of the sympatho-parasympathetic antagonism on the field: contemporary reviews span the range from more or less explicit acknowledgments that thoracolumbar sympathetic neurons are *“*involved in erection” {[45] (p 357), [52] (p 29)}, to elaborate dismissals of this pathway as a plasticity phenomenon induced by lesions of the spinal cord [50] or protests that “under normal conditions, stimulation of pelvic splanchnic nerves (PSN; nervi erigentes, parasympathetic) and of the hypogastric nerve (sympathetic) have different effects on erectile tissues” [53]. Somehow, the lumbosacral synergy is less controversial concerning the emission phase of ejaculation (“nicely integrated” [50] since “both sympathetic and parasympathetic tones act in a synergistic fashion to initiate seminal emission” [54] and [45] (p. 357) and references therein)—and the ejaculation center is lumbar [55, 56], so that the command of the male sexual act has come to be understood as shifting seamlessly, and oddly, from start to finish, from “parasympathetic” to sympathetic neurons [50].

In other words, and barring the residual controversy about erection, the notion of antagonism, which inspired, in the first place, the partition of the spinal cord into sympathetic and parasympathetic [2] has all but evaporated. In conclusion, we can only concur with Jänig that “functions assumed to be primarily associated with sacral […] systems are well duplicated by thoracolumbar […] pathways” and that the “division of the spinal autonomic systems into sympathetic and parasympathetic with respect to sexual function is questionable” [45] (p. 357).

## Conclusion

We have argued that the classification of the sacral autonomic outflow as sympathetic rather than parasympathetic on an ontogenetic criterion not only does not clash with any strong anatomical or physiological argument for an opposite view, but better fits many anatomical and physiological data. Gaskell was a staunch advocate of the deep connection between physiology, embryonic development and evolution, and his texts are peppered with speculations about the latter two. Langley himself concluded his statement, cited above, on the contrasted effects of lumbar and sacral nerves on blood vessels by “thus it seems to me probable that in the evolution of mammals the sympathetic nerves have developed at one time and the cranial and sacral involuntary nerves have developed at another time” [2]. One could argue that our reinterpretation of the sacral outflow in the light of embryonic development—and by implication evolution—is a continuation of Gaskell’s and Langley’s vision.

Apart from the sheer inertia of dogmas, what has allowed the wrong “parasympathetic” label to stick to the sacral outflow for so long might be that pelvic functions (storage and voiding) are not clearly homeostatic or easily comparable to the action of other viscera. Getting rid of the imaginary sympatho–parasympathetic complexity in that region will hopefully open the way to deciphering its real complexity: the diversity of cell types in the pelvic sympathetic system, both centrally and peripherally (including its antagonistic vasodilators and vasoconstrictor neurons), some of which we expect to be distributed across the former sympatho/“parasympathetic” divide, with the help of single cell transcriptomics (as in [57]) and novel techniques for the mapping of fine-grained connectivity [58].

## Footnotes

1 Our translation.

2 Our translation.

3 Our translation.
